# Supplementary material for: Numerical simulation of a hot-air cleaning fan for the combine harvester
Source: PLoS One. 2026 Mar 23;21(3):e0344780. doi: 10.1371/journal.pone.0344780 (PMC13008064; doi:10.1371/journal.pone.0344780)
Supplement: S1 Table — (PDF) [file pone.0344780.s001.pdf]

Grid No.	Average air velocity of upper outlet(m/s)	Average air velocity of lower outlet(m/s)
grid1	7.3067	4.9552
grid2	7.3798	4.9995
grid3	7.4036	5.0365
grid4	7.4815	5.0869
