# Supplementary material for: Numerical simulation of a hot-air cleaning fan for the combine harvester
Source: PLoS One. 2026 Mar 23;21(3):e0344780. doi: 10.1371/journal.pone.0344780 (PMC13008064; doi:10.1371/journal.pone.0344780)
Supplement: S2 Table — (PDF) [file pone.0344780.s002.pdf]

	Average air velocity (m/s)	
Z	Circular inlet fan	Modified inlet fan
0	15.11	13.14
0.144	15.56	13.36
0.288	16.36	13.92
0.432	17.88	14.99
0.576	22.56	20.88
0.72	10.35	11.77
