# Supplementary material for: Numerical simulation of a hot-air cleaning fan for the combine harvester
Source: PLoS One. 2026 Mar 23;21(3):e0344780. doi: 10.1371/journal.pone.0344780 (PMC13008064; doi:10.1371/journal.pone.0344780)
Supplement: S3 Table — (PDF) [file pone.0344780.s003.pdf]

	Average air velocity (m/s)		
Outlet	Circular fan	Modified fan	Actual fan
1	11.21	9.15	9.56
2	5.38	3.12	3.46
3	7.35	5.63	6.23
4	12.3	9.9	10.35
