# Supplementary material for: Numerical simulation of a hot-air cleaning fan for the combine harvester
Source: PLoS One. 2026 Mar 23;21(3):e0344780. doi: 10.1371/journal.pone.0344780 (PMC13008064; doi:10.1371/journal.pone.0344780)
Supplement: S4 Table — (PDF) [file pone.0344780.s004.pdf]

	air velocity (m/s)				air temperature (℃)			
Outlet transverse length (mm)	outlet 1	outlet 2	outlet 3	outlet 4	outlet 1	outlet 2	outlet 3	outlet 4
0	11.26	4.53	8.82	8.26	66.62	25.63	73.95	72.63
140	9.82	4.6	8.53	11.37	65.89	24.87	66.56	68.45
280	8.53	5.67	3.53	10.53	66.54	46.62	55.6	65.23
420	10.56	9.16	1.53	10.38	65.95	52.57	59.64	63.79
560	10.33	7.87	2.52	10.33	66.32	53.68	24.37	62.23
700	13.12	5.82	2.61	11.64	59.68	47.33	24.85	51.59
840	10.43	4.59	3.2	11.34	59.82	46.98	25.21	54.65
980	12.06	4.87	2.82	10.07	64.97	47.53	25.37	59.26
1120	8.44	7.68	1.27	10.33	65.56	56.63	29.34	66.35
1260	10.45	4.03	7.45	10.89	66.34	25.34	66.49	69.55
1400	11.32	3.32	8.63	9.47	66.89	25.67	72.83	72.49
