# Supplementary material for: Numerical simulation of a hot-air cleaning fan for the combine harvester
Source: PLoS One. 2026 Mar 23;21(3):e0344780. doi: 10.1371/journal.pone.0344780 (PMC13008064; doi:10.1371/journal.pone.0344780)
Supplement: Attachment — Submitted filename: Response to Reviewers.docx [file pone.0344780.s006.docx]

Dear Editor and Reviewers:

On behalf of my co-authors, we are very grateful to you for giving us an opportunity to revise our manuscript. we appreciate you very much for your positive and constructive comments entitled “Numerical simulation of a hot-air cleaning fan for the combine harvester”.

We have studied reviewers’ comments carefully and tried our best to revise our manuscript according to the comments. The following are the responses and revisions I have made in response to the reviewers' questions and suggestions on an item-by-item basis. Thanks again to the hard work of the editor and reviewer!

**Response to editor:**

1.The manuscript has been revised according to PLOS ONE's style requirements.

2.The sponsor is responsible for overseeing the grammar and formatting of the paper during the study.

3.I have removed the funding statement from my paper and would like to update it through the online submission form. The updated statement will be“Funding: This research was funded by National Natural Science Foundation of China under Grant grant number (51975257). ”

5. Data Availability Statement:All relevant data are within the manuscript. Minmal data set ：

Fig5.

| Grid No. | Average air velocity of upper outlet(m/s) | Average air velocity of lower outlet(m/s) |
| --- | --- | --- |
| grid1 | 7.3067 | 4.9552 |
| grid2 | 7.3798 | 4.9995 |
| grid3 | 7.4036 | 5.0365 |
| grid4 | 7.4815 | 5.0869 |

Fig9.

|  | Average wind pressure (pa) | |
| --- | --- | --- |
| Z | Circular inlet fan | Modified inlet fan |
| 0 | -107.149 | -116.39 |
| 0.144 | -110.781 | -126.55 |
| 0.288 | -119.279 | -138.86 |
| 0.432 | -144.769 | -152.95 |
| 0.576 | -171.618 | -179.9 |
| 0.72 | -51.0718 | -97.56 |

Fig10.

|  | Average air velocity (m/s) | |
| --- | --- | --- |
| Z | Circular inlet fan | Modified inlet fan |
| 0 | 15.11 | 13.14 |
| 0.144 | 15.56 | 13.36 |
| 0.288 | 16.36 | 13.92 |
| 0.432 | 17.88 | 14.99 |
| 0.576 | 22.56 | 20.88 |
| 0.72 | 10.35 | 11.77 |

Fig12.

|  | Average air velocity (m/s) | | |
| --- | --- | --- | --- |
| Outlet | Circular fan | Modified fan | Actual fan |
| 1 | 11.21 | 9.15 | 9.56 |
| 2 | 5.38 | 3.12 | 3.46 |
| 3 | 7.35 | 5.63 | 6.23 |
| 4 | 12.3 | 9.9 | 10.35 |

Fig23.

| Outlet | Simulated average air velocity(m/s) | Measured average air velocity(m/s) | Simulated average air temperature(℃) | Measured average air temperature(℃) |
| --- | --- | --- | --- | --- |
| 1 | 9.15 | 9.56 | 68.78 | 66.43 |
| 2 | 3.12 | 3.46 | 32.16 | 30.35 |
| 3 | 5.63 | 6.23 | 47.36 | 42.55 |
| 4 | 9.9 | 10.35 | 66.6 | 64.15 |

Fig28.

|  | air velocity (m/s) | | | | air temperature (℃) | | | |
| --- | --- | --- | --- | --- | --- | --- | --- | --- |
| Outlet transverse length  (mm) | outlet 1 | outlet 2 | outlet 3 | outlet 4 | outlet 1 | outlet 2 | outlet 3 | outlet 4 |
| 0 | 11.26 | 4.53 | 8.82 | 8.26 | 66.62 | 25.63 | 73.95 | 72.63 |
| 140 | 9.82 | 4.6 | 8.53 | 11.37 | 65.89 | 24.87 | 66.56 | 68.45 |
| 280 | 8.53 | 5.67 | 3.53 | 10.53 | 66.54 | 46.62 | 55.6 | 65.23 |
| 420 | 10.56 | 9.16 | 1.53 | 10.38 | 65.95 | 52.57 | 59.64 | 63.79 |
| 560 | 10.33 | 7.87 | 2.52 | 10.33 | 66.32 | 53.68 | 24.37 | 62.23 |
| 700 | 13.12 | 5.82 | 2.61 | 11.64 | 59.68 | 47.33 | 24.85 | 51.59 |
| 840 | 10.43 | 4.59 | 3.2 | 11.34 | 59.82 | 46.98 | 25.21 | 54.65 |
| 980 | 12.06 | 4.87 | 2.82 | 10.07 | 64.97 | 47.53 | 25.37 | 59.26 |
| 1120 | 8.44 | 7.68 | 1.27 | 10.33 | 65.56 | 56.63 | 29.34 | 66.35 |
| 1260 | 10.45 | 4.03 | 7.45 | 10.89 | 66.34 | 25.34 | 66.49 | 69.55 |
| 1400 | 11.32 | 3.32 | 8.63 | 9.47 | 66.89 | 25.67 | 72.83 | 72.49 |

6. I have an ORCID iD and that it has been validated in Editorial Manager.
7. The reviewer comments do not include a recommendation to cite specific previously published works.

**Response to reviewer:**

**Comment no.1：**Literature review is not up to date.

**Response：**As for the problem of outdated references you mentioned, we have carefully checked and updated them. The newly added references will better support our research arguments and enable our study to be more effectively connected with and compared to previous works. See lines 85-95.

**Comment no.2**：Need for the present study is not clearly spelt out.

**Response：**Thank you for your valuable advice,the need for the present study has been added in the article, see lines 120-127.

**Comment no.3：**Governing equations invoked is not specified

**Response：**Thank you for your valuable advice,The governing equations invoked have been specified. See line 216，eqs 9-11.

**Comment no.4：**Grid independence study not available

**Response：**Thank you for your valuable advice, the grid independence study is presented in section 3.3, line 234.

**Comment no.5**：Turbulence models study not done

**Response：**Thank you for your valuable advice,Turbulence model of k-epsilon was added from Eqs12 and 13,see line 220.

**Comment no.6：**Detail discussion on the findings between the improvement observed in the current and modified models not clearly specified

**Response：**Thank you for your valuable advice,Detail discussion on the findings between the improvement observed in the current and modified models has been added in the Sec 9，line637-639. The details are“In the present investigation, discrepancies between the model and the actual fan yield variances between simulation and test results. As shown in Fig 12 and Fig 23, the air velocity of the circular inlet fan and actual fan maximum discrepancies 18%. The maximum discrepancies of air velocity between the modified fan and the actual fan is 8.7%. The discrepancy in the error between the two types of fans arises from the model's structure.By comparing the simulation results with the test results, although the air velocity of the outlet of the improved fan is smaller than that of the circular inlet fan, it is closer to the air velocity of the actual fan. The simulated experimental conditions tend to be more idealistic than the actual experimental conditions.”

**Comment no.7：**Conclusion to be rewritten with focus on the current findings

**Response：**Thank you for your valuable advice,the Conclusion has been rewritten.The details are“In an endeavor to enhance the cleaning efficiency of the combine harvester, this study introduced a novel hot-air cleaning fan. Through an integration of FLUENT 2021 simulation analysis and experimental results from Design Expert 13.0, it was determined that the modified hot-air cleaning fan demonstrates superior performance. Based on the aforementioned research outcomes, the following primary conclusions were derived:

(1)By comparing the simulation and actual performance differences between the round and improved inlet fans, it was determined that the improved inlet structure could more accurately simulate the actual airflow characteristics of the fan and provide a reliable model basis for subsequent optimization.

(2)The outlet temperature of the fan was most significantly affected by the inlet temperature, and less affected by the volute temperature. The outlet wind speed was mainly determined by the rotation speed, and was positively correlated.

(3)The ideal operating parameter combination of the heating fan was determined using the response surface approach, which is 1445 rpm, 85 °C for the input temperature, and 60 °C for the volute temperature. The optimization was confirmed by bench tests, which produced an outlet velocity of 4.63–10.57 m/s and a temperature of 41.16–64.96 °C, both of which satisfied the requirements for wet crop separation. ”
