# Supplementary material for: Numerical simulation of a hot-air cleaning fan for the combine harvester
Source: PLoS One. 2026 Mar 23;21(3):e0344780. doi: 10.1371/journal.pone.0344780 (PMC13008064; doi:10.1371/journal.pone.0344780)
Supplement: Attachment — Submitted filename: Response_to_Reviewers_auresp_2.docx [file pone.0344780.s007.docx]

Dear Editor and Reviewers:

On behalf of my co-authors, we are very grateful to you for giving us an opportunity to revise our manuscript. we appreciate you very much for your positive and constructive comments entitled “Numerical simulation of a hot-air cleaning fan for the combine harvester”.

We have studied reviewers’ comments carefully and tried our best to revise our manuscript according to the comments. The following are the responses and revisions I have made in response to the reviewers' questions and suggestions on an item-by-item basis. Thanks again to the hard work of the editor and reviewer!

**Response to editor:**

1. The manuscript requires substantial revision for standard academic English, including grammar and sentence structure.

**Response：**The grammar and sentence structure of this manuscript have been revised .

2. The overall structure of the manuscript should be revised, including consolidation of multiple “results and discussion” and “conclusion” sections into single, appropriately organized sections。

**Response：**The multiple “results and discussion” and “conclusion”sections have been consolidated into single.

3. All equations taken or adapted from previous literature (including Equations 1–5) must be properly cited, and this should be ensured consistently throughout the manuscript.

**Response：**All equations taken or adapted from previous literature have been properly cited.

4. The logical relationship between CFD simulation, bench testing, and response surface methodology should be clearly explained and justified.

**Response：**The logical relationship between CFD simulation, bench testing, and response surface methodology has been clearly explained and justified.

5. The presentation of verification results should be clarified, and overlapping sections should be merged where appropriate.

**Response：**The presentation of verification results has been clarified, and overlapping sections have been merged where appropriate.

6. The conclusion section should be revised to be more concise and to emphasize quantitative findings.

**Response：**The conclusion section has been revised to be more concise and the quantitative findings have been emphasized.

7. Table 3 should be reformatted to comply with PLOS ONE table guidelines.

**Response：**The format of Table 3 has been revised.

8. The literature review should be strengthened, including the incorporation of more recent relevant studies.

**Response：**The literature review has been revised and more recent relevant studies have been cited.

**Response to reviewer2:**

**Comment no.1：**The manuscript does not adhere to standard English structures. A thorough revision is necessary to edit the structures.

**Response：**Thank you for your valuable advice, the grammar and sentence structure of this manuscript have been revised .

**Comment no.2**：The article is lengthy, and this volume is somewhat too extensive for publication in the journal. Please condense the content to make it more concise and effective.

**Response：**Thank you for your valuable advice, the content of this article has been condensed.

**Comment no.3：**Please provide the references for each of the equations (1 to 5) that you have taken from other sources. Additionally, please ensure this for all other equations as well.

**Response：**Thank you for your valuable advice,all the references for each of the equations that taken from other sources have been provided, such as equations 9-13,see line 121 and 125.

**Comment no.4：**Why have terms such as "results and discussion" and "conclusion" been used multiple times in different sections, like 3.3, 5, 9, and 10? This format is not suitable for publication in a reputable journal like PLOS ONE. Every article should have a single conclusion, so please review and revise the entire paper from the beginning。

**Response：**Thank you for your valuable advice, the multiple “results and discussion” and “conclusion”sections have been consolidated into single.

**Comment no.5**：The format of Table 3 is not suitable for publishing in this journal

**Response：**Thank you for your valuable advice, the format of Table 3 has been revised, see line 256.

**Response to reviewer3:**

**Comment no.1：**The structure of the introduction needs to be modified, there is no need to divide it into so many paragraphs. In addition, the content in the last paragraph, from line 102 to line 106, starting with "in conclusion", is unnecessary and not suitable for writing in the introduction.

**Response：**Thank you for your valuable advice,the structure of the introduction has been condensed and the content in the last paragraph has been revised.

**Comment no.2：**I agree with the previous reviewer's opinion that the literature review is still very insufficient. Currently, the cited literature is too old, mostly published before 2015. Please review the literature published in the past five years and add it to the manuscript. Otherwise, the current literature review cannot reflect the necessity and innovation of your research。

**Response：**Thank you for your valuable advice,the literature review has been revised and more recent relevant studies have been cited.

**Comment no.3：**Section 4.2, where is the results of the verification ? Is it included in Section 6? If so, the two should be merged.

**Response：**Thank you for your valuable advice, the results of the verification is included in section 6 and the two have be merged in section 5.

**Comment no.4：**Please the authors to explain the logical relationship between CFD simulation, bench testing, and response surface methodology analysis ? Why is it necessary to use all three methods simultaneously in one research ?

**Response：**Thank you for your valuable advice. Firstly, the simulation provides a theoretical basis for the design of the inlet structure of the hot airflow fan. Then, the working parameters are optimized through the response surface method. Finally, the simulation results were verified through the bench test. All three methods were used together in this study, forming a coherent chain from theoretical analysis to experimental validation.

**Comment no.5：**The conclusion is still too long and lacks quantitative results.

**Response：**Thank you for your valuable advice,the conclusion section has been revised to be more concise and the quantitative findings have been emphasized.
